# Providing data analytic services for national knowledge users in a federated system: learnings from two Canadian use cases

**DOI:** 10.23889/ijpds.v9i5.2890

**Published:** 2024-09-18

**Authors:** Raquel Duchen, Luke Mondor, Jodi Gatley, Refik Saskin, Jeff Bakal, Ted McDonald, John Knight, Nathan Nickel, Charles Burchill, Erik Youngson, Sandra Magalhaes, Andrew Goosen, Sonya Bowen, Lisa Flaten, Natalie Troke, Christine Warren, Zoe Hsu, Xueyi Chen, Lihui Liu, Yan Wang, Chandy Somayaji, Linyun Shen, Simon Youssef, Sarah Magee, Daniel Dutton

**Affiliations:** 1 HDRN Canada; 2 ICES; 3 Alberta Health Services; 4 New Brunswick Institute for Research, Data and Training (NB-IRDT), UNB; 5 Newfoundland and Labrador Health Services; 6 Manitoba Centre for Health Policy; 7 Canadian Institute for Health Information (CIHI); 8 Health Data Nova Scotia, Dalhousie University; 9 Maritime SPOR Support Unit, Nova Scotia Health Authority; 10 Canadian Institute for Health Information (CIHI)

## Objective and Approach

Canada’s federated health data system along with access pathways geared toward academic research, pose challenges for knowledge Users (KUs) requiring timely, pan-Canadian evidence to inform decisions. To understand challenges and explore solutions, we conducted two use cases for a pan-Canadian health technology assessment organization and a federal agency, using health administrative data at one federal and six provincial data centres. The population-based cohort studies described socio-demographics, comorbidities, treatment patterns, service utilization and costs. One focused on spinal muscular atrophy, a rare disease and the other on dementia, a complex chronic disease.

## Results

Administrative challenges included aligning varied ethical review and data access policies/procedures including requiring local and/or academic principal investigators and differing definitions of “research” vs. planning, evaluation, and monitoring. Data-related challenges included differences in structure, timeliness, and completeness across regions resulting in difficulty aligning constructs such as incident cases and episodes of care. Privacy requirements prohibited pooling jurisdictional estimates resulting in “small cells” that couldn’t be shared with KUs.

## Conclusions

We provided analytic outputs from multiple regions, albeit with some differences and gaps, increasing knowledge around both diseases while developing capacity for combined analyses and gaining insight into national possibilities for data access.

## Implications

As decision makers must rely on best available information, some data is better than none.  However, to improve data-analytic services for Pan-Canadian KUs, next steps will include improving data harmonization, expanding data assets and filling data gaps, implementing common data models, and exploring options for federated and/or pooled analyses.

